# Current tuberculosis status and challenges among dialysis patients in Japan: A nationwide cross-sectional survey

**DOI:** 10.1097/MD.0000000000044903

**Published:** 2025-10-31

**Authors:** Ayumi Yoshifuji, Kan Kikuchi, Ryoichi Ando, Yuki Uehara, Yoshihiko Kanno, Toru Kawai, Naoto Masuda, Keita Morikane, Tomoya Nishino, Ken Sakai, Yaoko Takano, Munekazu Ryuzaki, Shu Wakino, Yoshiaki Takemoto, Tadashi Tomo

**Affiliations:** a Infection Control Committee, The Japanese Society for Dialysis Therapy, Tokyo, Japan.

**Keywords:** dialysis, interferon-gamma release assay screening, latent tuberculosis infection, tuberculosis, tuberculosis transmission

## Abstract

Since 2021, Japan has been classified as a country with a low prevalence of tuberculosis (TB). Although dialysis patients have significantly higher incidences of TB and mortality, no recent nationwide surveys have assessed the TB status among dialysis patients in Japan. We conducted a nationwide cross-sectional survey of all 4167 dialysis facilities registered with the Japanese Society for Dialysis Therapy and collected facility-level data between April 2022 and March 2024. The survey assessed TB diagnoses (latent tuberculosis infection [LTBI] and active TB), diagnostic triggers, screening practices and treatment rates for LTBI, epidemiological data and clinical characteristics of active TB, incidence of TB close contacts, healthcare providers’ perceptions of TB in dialysis patients, and challenges associated with managing TB in dialysis patients. Survey responses were received from 2468 facilities with 194,720 dialysis patients. Among these patients, 331 were diagnosed with LTBI and 196 developed active TB during the 2-year study period. The LTBI rate was 85.0 cases per 100,000 person-years (95% confidence interval: 75.8–94.2), and the estimated annual rate of active TB was 50.3 cases per 100,000 person-years (95% confidence interval: 43.3–57.5). LTBI was most frequently diagnosed during screening before dialysis initiation (42.7%); however, only 4.5% of facilities conducted routine interferon-gamma release assay screening. The LTBI treatment rate was 75.2%. Among patients with active TB, diabetic nephropathy was the most prevalent underlying disease (44.2%); additionally, extrapulmonary TB was frequently observed. The incidence of active TB was highest among patients approximately 70 years of age (40.2%). Furthermore, 22.2% of patients developed TB within 3 months after dialysis initiation and 61.6% developed TB more than 1 year later. The mortality rate of these patients was 28.8%. Concerningly, 43.2% of dialysis healthcare staff appeared unaware of the TB risk, and 63.8% of facilities reported challenges regarding patient transfer and care coordination. TB is a significant burden among dialysis patients in Japan. Therefore, early awareness and treatment of TB, standardized LTBI screening protocols, robust treatment infrastructure with enhanced awareness among healthcare providers, as well as strengthened collaboration between medical institutions, government agencies, and TB-specialized healthcare facilities are necessary.

**Key points**

• The incidence of tuberculosis is elevated among dialysis patients, especially in the early post-dialysis initiation phase.
• Latent tuberculosis infection screening is limited despite its risk.
• Enhanced awareness and coordination among medical facilities are essential.

## 1. Introduction

In 2021, Japan was officially classified as a country with a low tuberculosis (TB) prevalence for the first time. By 2023, the national TB incidence rate had declined to 8.1 cases per 100,000 persons.^[1]^ However, a systematic review that assessed the risk of active TB among dialysis patients reported that this subpopulation exhibited a 7.69-fold higher risk of TB than that of the general population because of impaired cellular immunity.^[2]^

Dialysis patients with TB present with several unique challenges. TB is more frequently diagnosed within the 1st year of dialysis initiation and is often characterized by extrapulmonary manifestations such as lymph node TB and miliary TB. Additionally, treatment options for such patients are limited, resulting in a poor prognosis and increased difficulty associated with disease management.^[3,4]^ In Japan, dialysis patients who have smear-positive TB results require isolation during dialysis sessions, and many affected individuals are older adults with prolonged hospital stays and the declining ability to perform activities of daily living, resulting in particularly challenging patient admissions. Furthermore, if patients actively shed TB bacteria, then the enclosed environment of dialysis units leads to a high number of close contacts, thereby increasing the risk of TB transmission.^[3]^

To address these concerns, the World Health Organization recommends the early diagnosis and treatment of latent tuberculosis infection (LTBI) for this patient subpopulation before progression to active TB.^[5]^ The interferon-gamma release assay (IGRA) has been recognized as a useful tool for diagnosing LTBI, and its effectiveness has been demonstrated for dialysis patients.^[6]^ In Japan, the Guidelines for Standard Hemodialysis Procedure and Prevention of Infection in Maintenance Hemodialysis Facilities (6th edition) recommend treatment for these patients diagnosed with LTBI.^[7]^ However, these guidelines do not provide specific recommendations for LTBI screening, and IGRA screening is not covered by the Japanese healthcare insurance system.

According to Kimura et al, 17.9% of patents who initiated dialysis were IGRA-positive.^[8]^ Additionally, Ogawa et al reported that 11.9% of outpatients on maintenance dialysis were IGRA-positive.^[9]^ However, few nationwide studies of TB and LTBI in dialysis patients have been conducted in Japan. Because of this lack of data, the Infection Control Committee of the Japanese Society for Dialysis Therapy (JSDT) conducted a nationwide survey to clarify the current epidemiology of TB among hemodialysis patients, identify key challenges associated with TB control and management, assess the status of LTBI screening and treatment among patients initiating dialysis, and evaluate the significance of the LTBI diagnosis and treatment among this high-risk population. This survey aimed to provide a foundation to reduce TB transmission and propose appropriate strategies to improve TB prevention and management.

## 2. Methods

In May 2024, the Current Status of Tuberculosis in Dialysis Patients survey was distributed to 4 167 dialysis facilities that were registered as members of the JSDT. These facilities were asked to respond to this survey via mail or the Internet (Supplemental Digital Content 1, Supplemental Digital Content, https://links.lww.com/MD/Q281). This survey collected data regarding TB diagnoses, including cases of LTBI and active TB, at the respondent facilities between April 1, 2022 and March 31, 2024. The survey was designed to collect aggregate facility-level data. Each facility was instructed to submit 1 response, and completion by a physician or dialysis healthcare staff member was preferred. All responses were anonymous, and individual patient-level information and personal identifiers were not collected.

The questionnaire comprised 5 main sections. Section 1 gathered facility characteristics, including geographic location, number of dialysis beds, availability of isolation capabilities, and whether IGRA screening was conducted for new or transferred patients. Section 2 collected the total number of patients diagnosed with LTBI and active TB during the 2-year study period as well as their summarized characteristics, including clinical presentation, age distribution, underlying renal disease (e.g., diabetic nephropathy), time from dialysis initiation to TB onset, and treatment outcomes. Section 3 addressed the occurrence of TB contacts within dialysis facilities and included a brief item about whether screening-related costs were covered by the facility. Section 4 asked respondents whether they perceived dialysis patients as being at higher risk for TB based on their clinical experience. Section 5 was an open-ended item. Respondents were invited to describe any challenges or difficulties encountered during the management of TB in dialysis patients. Depending on the function of the facility (outpatient, inpatient, or both), different subsections were provided to ensure relevance and minimize respondent burden. According to the guidelines of the JSDT, because this study involved anonymous aggregate facility-level data and did not include individual patient information, formal ethical approval was not required.

### 2.1. Statistical analysis

The diagnosis rate of LTBI and incidence rate of active TB were calculated by dividing the number of reported cases by the total number of dialysis patients at the respondent facilities during a 2-year period (April 1, 2022–March 31, 2024). Then, annual incidence rates per 100,000 person-years were computed using the total number of person-years (the total number of dialysis patients at the respondent facilities × 2 person-years). Patients initially diagnosed with LTBI who subsequently developed active TB were included as active TB cases. The 95% confidence intervals (CIs) of incidence rates were calculated assuming a Poisson distribution. To examine factors associated with the implementation of LTBI screening using the IGRA, we categorized responses as follows: answers of “yes” or “based on risk” indicated that IGRA screening was implemented (screening present), and answers of “no” indicated that IGRA screening was not implemented (screening absent). Logistic regression was used to compare implementation rates across geographic regions, facility types (hospital or clinic), and number of patients on maintenance dialysis. Furthermore, to evaluate regional and facility-level variations in the TB incidence, we performed Poisson regression using the number of active TB cases as the outcome, and region, facility type, and number of patients on maintenance dialysis were used as predictors. When applicable, descriptive statistics such as the mean, median, and interquartile range were used to summarize survey results. All analyses were performed using IBM SPSS Statistics for Windows (version 28.0; IBM Corp., Armonk) and Microsoft Excel 2019 (Microsoft Corp., Redmond).

## 3. Results

**Figure 1.**
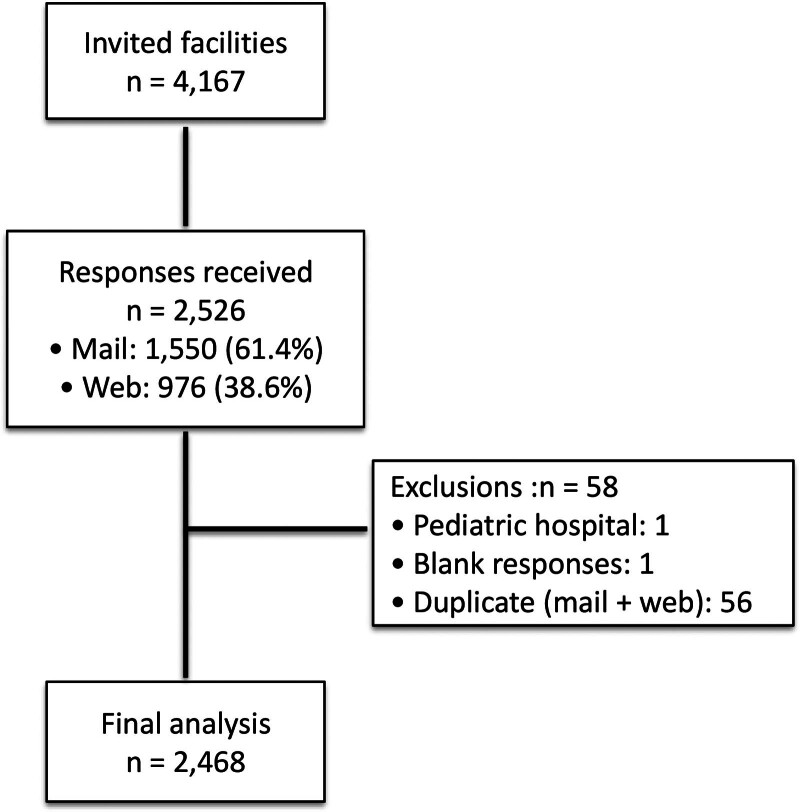
Flowchart of facility inclusion. The inclusion process for dialysis facilities in the nationwide tuberculosis (TB) survey conducted in Japan between April 2022 and March 2024. Among 4167 facilities invited to participate, 2526 facilities responded. After excluding 58 facilities because of pediatric-only practices, incomplete responses, or duplicate submissions, 2468 facilities were included in the final analysis.

Of the 4167 facilities surveyed, 2526 (60.6%) responded. Of these responses, 1550 (61.4%) were received via mail and 976 (38.6%) were received via the Internet. After excluding 58 duplicate responses, 2468 (59.2%) valid responses were included in the final analysis (Fig. 1). The included facilities collectively included a total of 194,720 dialysis patients; this number was used as the denominator when calculating incidence rates.

### 3.1. Facility characteristics

The regional distributions of the responding facilities (n = 2455) and their types (2468 facilities with valid responses) are available online (Supplemental Digital Content 2, Supplemental Digital Content, https://links.lww.com/MD/Q281). Among the 1093 responding inpatient clinics and hospitals, 200 (18.3%) reported the ability to provide inpatient treatment for patients with smear-positive TB results.

### 3.2. Current status of the TB diagnosis

Among the 2463 facilities that provided responses regarding the current status of the TB diagnosis, 267 (10.8%) reported at least 1 TB diagnosis. The number of patients diagnosed with LTBI was 331 (0.13 ± 0.82 patients per facility), and the number of patients with active TB was 196 (0.08 ± 0.34 patients per facility). The estimated annual incidence of active TB was 50.3 cases per 100,000 person-years (95% CI: 43.3–57.5), and that of LTBI was 85.0 cases per 100,000 person-years (95% CI: 75.8–94.2).

### 3.3. Latent TB infection

The regional distribution of LTBI cases is presented online (Supplemental Digital Content 3, Supplemental Digital Content, https://links.lww.com/MD/Q281). LTBI was most commonly diagnosed by screening performed at the time of dialysis initiation (42.7%) and as a differential diagnosis for lung abnormalities (20.2%) (Table 1). According to the questionnaire responses, only 200 (75.2%) of 266 patients diagnosed with LTBI received treatment.

**Table 1:** Diagnostic triggers for LTBI.

	n	%
Screening at the time of dialysis initiation	137	42.7
Differential diagnosis for abnormal pulmonary shadows	65	20.2
Differential diagnosis for fever and cough	42	13.1
Examination and diagnosis at another hospital	32	10
Contact with a tuberculosis patient	26	8.1
Risk of comorbid conditions	16	5
Routine examination	5	1.6
Screening test before kidney transplantation	2	0.6

Number of responses: 321.

LTBI = latent tuberculosis infection.

Regarding IGRA screening for LTBI in new and transferred dialysis patients, 110 (4.5%) facilities performed IGRA screening for all new patients; however, 418 (17.2%) facilities performed screening based on the risk assessment results (Table 2).

**Table 2:** LTBI screening practices at the time of dialysis initiation.

	n	%
Screening at the time of dialysis initiation	137	42.7
Differential diagnosis for abnormal pulmonary shadows	65	20.2
Differential diagnosis for fever and cough	42	13.1
Examination and diagnosis at another hospital	32	10
Contact with a tuberculosis patient	26	8.1
Risk of comorbid conditions	16	5
Routine examination	5	1.6

	n	%
No	1899	78.2
Dependent on risk	418	17.2
Yes	110	4.5

Number of responses: 2427.

LTBI = latent tuberculosis infection.

At the facilities that screened all new dialysis patients, 60 cases of LTBI were diagnosed during the 2-year study period. Based on the data of 1831 new dialysis patients, the estimated prevalence of LTBI was 3.3%.

According to the logistic regression analysis, IGRA screening was more frequently performed in Tokyo than in Shikoku (reference) (*P* = .038), in hospitals than in clinics (*P* < .001), and in facilities with 50 to 89 patients than in those with < 50 patients (*P* = .008) (Supplemental Digital Content 4, Supplemental Digital Content, https://links.lww.com/MD/Q281).

### 3.4. Current status of active TB

The survey identified 196 active TB cases in dialysis patients. The regional distribution of these cases is presented online (Supplemental Digital Content 5, Supplemental Digital Content, https://links.lww.com/MD/Q281). Moreover, a Poisson regression analysis of the TB incidence revealed that it was significantly higher in the Kinki region (*P* = .011) and Tokyo (*P* = .001) than in Chubu. Clinics had a significantly lower TB incidence than that of hospitals (*P* < .001). Facilities with 50 to 89 patients (*P* = .002) and those with ≥ 90 patients (*P* = .002) also had lower TB incidences than those of facilities with < 50 patients (Supplemental Digital Content 6, Supplemental Digital Content, https://links.lww.com/MD/Q281).

The most common underlying disease among dialysis patients diagnosed with TB was diabetic nephropathy (76 cases; 44.2%) (Table 3). Regarding the TB type at the time of onset, pulmonary TB was the most common form (74 cases; 39.2%); however, compared with nondialysis patients, dialysis patients exhibited a higher proportion of extrapulmonary TB, including tuberculous pleuritis, miliary TB, and lymph node TB.

**Table 3:** Clinical characteristics of dialysis patients with active tuberculosis.

Category	n	%
1. Primary indication for dialysis (n = 172)		
Diabetic nephropathy	76	44.2
Nephrosclerosis	37	21.5
Chronic glomerulonephritis	29	16.9
Others	30	17.4
2. Clinical presentation* (n = 189)		
Pulmonary tuberculosis	74	39.2
Tuberculous pleuritis	35	18.5
Miliary tuberculosis	28	14.8
Lymph node tuberculosis	21	11.1
Other tuberculosis type	32	16.9
3. Age at disease onset (n = 194)		
Younger than 40 yr	7	3.6
50–59 yr	12	6.2%
60–69 yr	30	15.5
70–79 yr	78	40.2
80–89 yr	53	27.3
90–99 yr	14	7.2
4. Duration from dialysis initiation to the TB diagnosis (n = 185)		
<3 mo	41	22.2%
3–6 mo	12	6.5%
6–12 mo	18	9.7%
>1 yr	114	61.6%
5. Prognosis (n = 191)		
Recovery (outpatient)	100	52.4%
Recovery (inpatient)	19	9.9%
Died as a result of another disease	33	17.3%
TB or TB-related death	22	11.5%
Undergoing treatment at another facility	10	5.2%
Undergoing treatment	5	2.6%
During admission	2	1.0%

TB = tuberculosis.

* Multiple answers were allowed in category 2.

The age distribution at the time of TB onset is detailed in Table 4. The highest incidence occurred in patients approximately 70 years of age (78 cases; 40.2%), although 25.3% of the cases occurred in patients younger than 60 years of age, thus highlighting that younger patients are not exempt from the risk of TB. Additionally, we compared age-specific TB incidence rates of dialysis patients with those of the general Japanese population, as reported by the 2023 Tuberculosis Surveillance Center.^[10]^ The TB incidence of dialysis patients was markedly higher than that of the general population across all age groups (Supplemental Digital Content 7, Supplemental Digital Content, https://links.lww.com/MD/Q281). Among those 70 to 79 years of age, the TB incidence was 60.0 cases per 100,000 person-years (95% CI: 48.5–71.5) for dialysis patients and 11.87 cases per 100,000 persons for the general population. This pattern was consistent, even among younger age groups. Dialysis patients younger than 50 years of age had a higher TB incidence than that of the general population (27.1 cases vs 17.4 cases per 100,000 persons), thus underscoring the heightened risk in this clinical population regardless of age.

**Table 4:** Facilities that reported TB close contacts during the 2-year study period.

	n	%
Patients	28	1.2
Medical staff	10	0.4
Patients and medical staff	39	1.6
No one	2351	96.8

Number of facilities that responded: 2428.

TB = tuberculosis.

The durations from dialysis initiation to TB onset are summarized in Table 3. For example, 22.2% of TB cases occurred within the 1st 3 months of dialysis initiation, thus emphasizing its early risk. However, 61.6% of patients developed TB more than 1 year after dialysis initiation, thus confirming that those on maintenance dialysis remained at risk for TB at any stage.

Finally, the prognostic data revealed that the mortality rate of TB patients was 28.8%. Twenty-two (11.5%) patients died as a result of TB or TB-related complications and 33 (17.3%) patients died as a result of other diseases, thus demonstrating the poor prognosis associated with TB in this patient subpopulation (Table 3).

### 3.5. Incidence of TB close contacts in dialysis facilities

The proportion of TB cases identified as close contacts by public health authorities during the 2-year study period is shown in Table 4. Among TB cases, 1.2% (28 cases) involved dialysis patients, 0.4% (10 cases) involved healthcare staff, and 1.6% (39 cases) involved both patients and healthcare staff.

Regarding the financial burden of contact screening, 111 (5.0%) patients incurred out-of-pocket expenses for IGRA screening and 86 (3.8%) patients incurred out-of-pocket expenses for chest X-ray screening at medical institutions (Table 5).

**Table 5:** Financial burden of contact screening on facilities.

	n	%
IGRA	111	5.0
Chest X-ray	86	3.8
Outpatient medical fee	17	0.8
None	2091	93.4

Number of facilities that responded: 2238.

IGRA = interferon-gamma release assay.

### 3.6. Perceptions of dialysis healthcare staff regarding the TB risk of dialysis patients

To assess awareness among dialysis healthcare staff, the survey included questions regarding their subjective perceptions of the TB risk of dialysis patients. Of the responding facilities, 43.2% indicated that they did not consider this subpopulation to be at increased risk for TB, suggesting their potential lack of awareness of TB susceptibility among these patients (Table 6).

**Table 6:** Perceived TB risk among dialysis healthcare providers.

	n	%
High risk	1116	47.1
Average risk	1024	43.2
Uncertain	217	9.2
Dependent on the patient’s condition and environment	13	0.5

Number of facilities that responded: 2370.

TB = tuberculosis.

### 3.7. Challenges managing TB in dialysis patients

Although TB is not uncommon among dialysis patients, ensuring smooth and effective treatment is a major challenge in clinical practice. The results of this survey highlighted “difficulty in arranging patient admission or transfer” as the most frequently reported issue (573 facilities; 63.8%) (Table 7). This finding underscores the critical barriers to TB management in dialysis settings and indicates the need for improved coordination among medical institutions to facilitate appropriate patient care.

**Table 7:** Challenges associated with managing TB in dialysis settings.

	n	%
Coordinating admissions and transfers	573	63.8
Concerns about isolation and infection control measures	39	4.3
Management of the contact person	29	3.2
Lengthy and difficult diagnostic process	28	3.1
Treatment management difficulties (including DOTS)	20	2.2
Insufficient knowledge of tuberculosis among medical staff	4	0.4
Complicated administrative procedures	3	0.3
Limited access to specialists for consultations	2	0.2

Number of facilities that responded: 698.

DOTS = directly observed treatment short-course.

## 4. Discussion

This survey successfully elucidated the current status of LTBI and active TB among dialysis patients in Japan and identified key challenges of TB management within this population.

In this study, 331 patients were diagnosed with LTBI and 196 patients developed active TB. Based on the number of dialysis patients at the facilities that responded to this survey (194,720 patients), the estimated TB diagnosis rate among dialysis patients was 135.3 cases per 100,000 persons. The estimated annual incidence of active TB was 50.3 cases per 100,000 person-years (95% CI: 43.3–57.5), and that of LTBI was 85.0 cases per 100,000 person-years (95% CI: 75.8–94.2). This rate was dramatically higher than that of the general Japanese population in 2023 (8.1 cases per 100,000 persons).^[1]^ Furthermore, this rate was similar to that of TB (146.0 cases per 100,000 dialysis patients) in a region of Tokyo in 2011.^[11]^

A systematic review reported that the risk of active TB among dialysis patients was 7.69-times higher than that of the general population, primarily because of impaired cellular immunity.^[2]^ In our study, we found that although the TB incidence was highest among patients 70 years of age or older, 25.3% of the cases occurred in patients younger than 70 years of age, indicating a definite risk among younger dialysis patients. We also calculated age-specific TB incidence rates among dialysis patients and compared them with data of the general Japanese population. Across all age groups, the incidence of TB in dialysis patients exceeded that in the general population, including those younger than 50 years of age (Supplemental Digital Content 7, Supplemental Digital Content, https://links.lww.com/MD/Q281). This suggests that the elevated TB risk in this population cannot be solely attributed to age; instead, it is attributed to factors specific to dialysis, such as impaired cellular immunity and treatment-related exposure. These findings further emphasize that targeted TB prevention strategies are necessary for dialysis patients, regardless of age.

TB in dialysis patients often presents with nonspecific symptoms; therefore, the diagnosis may be delayed because of low test sensitivity and specificity.^[4]^ Moreover, these patients who develop smear-positive pulmonary TB share treatment spaces with other patients, thereby increasing the risk of widespread infection transmission.^[7]^ Consequently, effective TB prevention measures are essential for this high-risk population. However, awareness of the high risk of TB in this subpopulation remains low at dialysis healthcare facilities, thus underscoring the urgent need for improved education and preventive measures (Table 6).

Furthermore, Takamori et al and Vikrant et al highlighted the high prevalence of extrapulmonary TB in this subpopulation.^[3,12]^ Our survey results corroborate these findings, thus revealing that although pulmonary TB accounted for 39.2% of cases, the proportion of extrapulmonary TB, including tuberculous pleuritis, miliary TB, and lymph node TB, was notably high.^[3,11]^

Previous research emphasized that the highest incidence of TB occurs within the first year of dialysis initiation, and particularly within the first 6 months of initiation.^[4,13]^ Our findings agree with those of the aforementioned studies and indicated that 28.7% and 22.2% of cases occurred within the first 6 months and within the first 3 months of dialysis initiation, respectively. However, a substantial proportion (61.1%) of TB cases developed more than first year after dialysis initiation, thus highlighting the need for continuous TB monitoring throughout the course of maintenance dialysis.

Mortality rates of dialysis patients with TB are concerning. Previous studies reported a mortality rate of 18.9% in Japan, whereas global data suggested mortality rates ranging from 14.3% to 36.8%.^[3,12]^ In our study, the TB-related mortality rate was 11.5%; however, when deaths from other causes were included, the overall mortality rate after TB onset reached 28.7%, thus demonstrating the poor prognosis of these patients. These findings underscore the importance of early diagnosis and treatment before TB progression.

The risk of progression from LTBI to active TB in dialysis patients is estimated to be 10- to 25-times higher than that in the general population.^[2]^ Therefore, early detection and treatment of LTBI are crucial. The IGRA has been identified as a highly effective diagnostic tool for LTBI with well-documented utility for these patients.^[6]^

Kimura et al estimated an LTBI prevalence of 17.9% among patients initiating dialysis,^[8]^ whereas Ogawa et al documented an LTBI prevalence of 11.9% among patients on maintenance dialysis.^[9]^ However, our survey found an LTBI prevalence of 3.3% among patients initiating dialysis, which was significantly lower than the previously reported rates. Although this may reflect the declining TB prevalence in Japan, the low prevalence in our study may have resulted from selection bias associated with surveying facilities that universally conduct IGRA screening.

Our survey further revealed that 4.5% of facilities performed LTBI screening for all new or transferred dialysis patients, whereas 17.2% implemented risk-based screening. The World Health Organization strongly recommends systematic TB screening (IGRA or tuberculin skin test) and preventive treatment for dialysis patients.^[5]^ In British Columbia, Canada, universal IGRA screening before dialysis initiation significantly reduced the incidence of active TB and was cost-effective.^[14]^

Conversely, the British Thoracic Society does not recommend universal TB screening for dialysis patients because of a lack of highly sensitive and specific diagnostic tools and insufficient data regarding the efficacy and safety of preventive treatment for TB.^[15]^

The JSDT Guidelines for Standard Hemodialysis Procedure and Prevention of Infection in Maintenance Hemodialysis Facilities (6th edition) recommend treating dialysis patients diagnosed with LTBI (level 1A).^[7]^ However, routine LTBI screening remains limited in Japan because of several factors. First, LTBI testing for asymptomatic patients is not reimbursed by the national health insurance system, which likely discourages facilities from adopting routine screening. Second, the JSDT Guidelines for Standard Hemodialysis Procedure and Prevention of Infection in Maintenance Hemodialysis Facilities (6th edition) have traditionally emphasized active TB case detection rather than preventive screening. Third, standardized implementation frameworks (such as standing orders [protocols that enable screening without individualized physician orders], electronic reminders [automated alerts in electronic medical records], and clinician education programs) are not widely established in Japan. These systemic limitations may contribute to the low screening rate (4.5%) observed in this study and suggest that policy-level support is essential to promoting targeted LTBI screening for high-risk groups such as dialysis patients. Aligning national practice with global recommendations may require not only updated clinical guidance but also reimbursement reform and enhanced institutional infrastructure.

The prevalence of LTBI (3.3%) among patients identified in this survey was comparable to that of new hepatitis C virus (3.1%) among Japanese dialysis patients.^[7]^ From the perspective of infection control, whether IGRA-based LTBI screening should be expanded despite the lack of insurance coverage requires further consideration. Dialysis patients are at substantially increased risk for progression from LTBI to active TB, particularly within the first year of initiating dialysis, when up to 22.2% of cases occur. Dissemination of these findings through nephrology and infectious disease societies is critical to raising awareness of both the risk of individual patients and the potential for rapid transmission in shared treatment spaces among dialysis staff. Of note, in British Columbia, Canada, where the incidence of TB is comparable to that in Japan, LTBI screening and treatment of dialysis patients have been demonstrated as not only clinically effective but also cost-effective by health economic evaluations. These findings support the notion that targeted LTBI screening of dialysis populations can be a justified strategy, even in countries with a declining TB prevalence, if appropriate infrastructure and programmatic support exist. However, robust local evidence is not yet available because such large-scale cohort studies and formal economic analyses have not yet been performed in Japan; therefore, nationwide implementation is challenging. Because of the overall declining TB incidence in Japan, universal screening may be difficult to justify at this stage. Therefore, a risk-based approach focused on older patients, those with low body weight or malnutrition, smokers, and ischemic heart disease patients may represent a more feasible and context-appropriate strategy for TB prevention in dialysis populations. Multicenter collaborative studies and cost-effectiveness evaluations are essential to determining the feasibility of LTBI screening in Japan.

Furthermore, 24.8% of patients with LTBI did not receive treatment, indicating suboptimal LTBI management. Improving awareness among Japanese medical societies regarding the high risk of TB for dialysis patients is crucial. Additionally, the lack of safety data regarding LTBI treatment for dialysis patients may be an impediment to its widespread adoption. Therefore, it is necessary to accumulate domestic safety data regarding LTBI treatment for dialysis patients.

Another key issue identified in this study is that some dialysis institutions still bear the financial burden of TB contact screening, thus highlighting the need for government collaboration to improve cost coverage. Moreover, when TB cases occur at dialysis facilities, the most frequently cited challenge is arranging hospital admissions and interfacility transfers. Addressing this issue requires coordinated efforts by government agencies to establish a structured TB referral system. Additionally, collaboration with respiratory and infectious disease societies is essential to ensuring efficient knowledge sharing and response preparedness.

This study provides comprehensive insights regarding the current epidemiology of LTBI and active TB among dialysis patients in Japan. However, some limitations of this study must be acknowledged. First, selection bias may have led to underestimation or overestimation of the true prevalence of TB because the nonresponding facilities (41%) may have differed systematically from the responding facilities. Second, our reliance on unvalidated self-reported facility data raised the possibility of underreporting, recall bias, and misclassification of LTBI and active TB cases, particularly in the absence of external validation (e.g., registry linkage or chart audits). Third, the cross-sectional survey design precluded accurate incidence estimation and detailed analyses of progression from LTBI to active disease. Fourth, we did not perform multivariable analyses to adjust for potential confounders (such as age, comorbidities, or facility characteristics), which may have influenced the TB risk estimates. Fifth, the perception-based question (“Based on your experience, do you think that dialysis patients are more prone to developing TB?”) was not derived from a validated psychometric instrument; therefore, it should be interpreted as a pragmatic measure of awareness rather than a formally tested construct. Finally, patient-level data (e.g., individual IGRA results, treatment adherence, and long-term outcomes) were unavailable, thus limiting our ability to assess screening effectiveness and treatment impact. To address these issues, future research should include registry-linked multicenter cohort studies with external data validation, standardized diagnostic criteria, and multivariate risk modeling to allow better characterization of the LTBI prevalence and progression risk among this vulnerable population.

## 5. Conclusion

This survey effectively elucidated the current TB burden among dialysis patients in Japan. Because of the poor prognosis associated with active TB in this population, standardizing LTBI screening protocols and establishing a comprehensive treatment framework are imperative. Additionally, to address the persistent challenge of securing hospital admissions for patients with smear-positive TB, enhanced collaboration between governmental authorities and specialized TB treatment facilities are crucial to ensuring timely and appropriate care.

## Acknowledgments

We thank all dialysis facilities for responding to the questionnaire survey.

## Author contributions

**Conceptualization:** Ayumi Yoshifuji, Kan Kikuchi.

**Data curation:** Ayumi Yoshifuji, Kan Kikuchi.

**Formal analysis:** Ayumi Yoshifuji, Kan Kikuchi.

**Funding acquisition:** Kan Kikuchi.

**Investigation:** Ayumi Yoshifuji, Kan Kikuchi.

**Methodology:** Ayumi Yoshifuji, Kan Kikuchi.

**Project administration:** Ayumi Yoshifuji, Kan Kikuchi.

**Resources:** Ayumi Yoshifuji.

**Supervision:** Kan Kikuchi.

**Validation:** Kan Kikuchi.

**Visualization:** Ayumi Yoshifuji, Kan Kikuchi.

**Writing – original draft:** Ayumi Yoshifuji, Kan Kikuchi.

**Writing – review & editing:** Ryoichi Ando, Yuki Uehara, Yoshihiko Kanno, Toru Kawai, Naoto Masuda, Keita Morikane, Tomoya Nishino, Ken Sakai, Yaoko Takano, Munekazu Ryuzaki, Shu Wakino, Yoshiaki Takemoto, Tadashi Tomo.

## Supplementary Material


