# Supplementary material for: Current tuberculosis status and challenges among dialysis patients in Japan: A nationwide cross-sectional survey
Source: Medicine (Baltimore). 2025 Oct 31;104(44):e44903. doi: 10.1097/MD.0000000000044903 (PMC12582689; doi:10.1097/MD.0000000000044903)
Supplement: Supplementary file 1 [file medi-104-e44903-s001.pdf]

Supplemental Digital Content 1: Questionnaire sheet

Survey on the Current Status of Tuberculosis in Dialysis Patients

Please provide responses for the period from April 1, 2022, to March 31, 2024.

1. Facility Overview

Please specify the prefecture where your facility is located.

Please enter the facility's phone number, including the area code. (This will be used to identify duplicate responses.)

■ Please select one facility type:

☐ Hospital ☐ Clinic with beds ☐ Clinic without beds ☐ Other

→ For clinics with beds and hospitals:

Can your facility provide inpatient treatment while tuberculosis (TB) bacteria are being excreted?

Please provide the number of beds available per dialysis session and isolated dialysis beds.

As of March 31, 2024, how many patients are on maintenance dialysis at your facility?

Are screening tests using the interferon-gamma release assay (IGRA) performed in new and transferred patients?

☐ Yes ☐ Conducted based on risk ☐ No

2. Tuberculosis patients for the Past Two Years (April 1, 2022–March 31, 2024)

■ In the past two years (April 1, 2022–March 31, 2024), have any maintenance dialysis patients at your facility been diagnosed with tuberculosis? (including patients diagnosed at other facilities)

☐ Yes ☐ No

→ If "Yes," please provide the number of patients diagnosed with latent tuberculosis

infection and the number who developed tuberculosis.

Latent tuberculosis infection cases: (patients); TB cases: (patients).

★ If your facility had patients with latent tuberculosis infection, please answer the following questions:

- What was the reason for the diagnosis and how many patients were diagnosed for each reason?

  ☐ Screening at dialysis initiation (patients)

  ☐ Considering the risk of comorbidities (patients)

  ☐ Differential diagnosis for fever (patients)

  ☐ Differential diagnosis for lung abnormal shadows (patients)

  ☐ Other (patients)

- How many patients diagnosed with latent tuberculosis infection received treatment? (patients)

★ If your facility had patients who developed tuberculosis, please answer the following questions:

If your facility is an outpatient dialysis facility, answer only section ①.

If your facility is an inpatient facility, answer only section ②.

If your facility provides both functions, answer section ① for outpatients and section ② for inpatients.

① Outpatient Maintenance Dialysis Facility

- If the patient was hospitalized for tuberculosis, please enter the hospital's phone number, including the area code.
- What type of tuberculosis develops? Please specify the number of cases for each condition:

  ☐ Pulmonary tuberculosis ☐ Tuberculous pleuritis ☐ Miliary tuberculosis ☐ Lymph node tuberculosis ☐ Other types of tuberculosis
- Please specify the number of tuberculosis patients by age group:

  ☐ ~40s ☐ 50s ☐ 60s ☐ 70s ☐ 80s ☐ 90+
- Please specify the number of tuberculosis patients by underlying disease:

  ☐ Diabetic nephropathy ☐ Chronic glomerulonephritis ☐ Nephrosclerosis ☐ Other ☐ Unknown
- Please specify the time from dialysis initiation to tuberculosis onset:

  ☐ Within 3 months ☐ Within 6 months ☐ Within 1 year ☐ More than 1 year
- Please specify the prognosis of tuberculosis patients:

  ☐ Recovered (Outpatient) ☐ Recovered (Inpatient dialysis) ☐ Died from other diseases ☐ Died due to tuberculosis or tuberculosis-related causes ☐ Other

② Inpatient Facility (Leave blank if no applicable patients)

- If there was an outpatient dialysis facility before hospitalization, please enter the phone number including the area code.
- What type of tuberculosis develops? Please specify the number of cases for each condition:

  ☐ Pulmonary tuberculosis ☐ Tuberculous pleuritis ☐ Miliary tuberculosis ☐ Lymph node tuberculosis ☐ Other types of tuberculosis

- Please specify the number of tuberculosis patients by age group:

  ☐ ~40s ☐ 50s ☐ 60s ☐ 70s ☐ 80s ☐ 90+

- Please specify the number of tuberculosis patients by underlying disease:

  ☐ Diabetic nephropathy ☐ Chronic glomerulonephritis ☐ Nephrosclerosis ☐ Other ☐ Unknown

- Please specify the time from dialysis initiation to tuberculosis onset:

  ☐ Within 3 months ☐ Within 6 months ☐ Within 1 year ☐ More than 1 year

- Please specify the prognosis of tuberculosis patients:

  ☐ Recovered (Outpatient) ☐ Recovered (Inpatient dialysis) ☐ Died from other diseases ☐ Died due to tuberculosis or tuberculosis-related causes ☐ Other

3. Tuberculosis Contacts in the Past Two Years

■ Were there any individuals who had contact with tuberculosis patients among dialysis patients?

☐ Patients ☐ Staff ☐ Both patients and staff ☐ None

■ Has your facility conducted screening tests for contacts at its own expense?

☐ IGRA test ☐ X-ray ☐ Outpatient consultation cost ☐ None

4. Tuberculosis in Dialysis Patients

Based on your experience, do you think that dialysis patients are more prone to developing tuberculosis?

☐ Yes ☐ No ☐ Other

5. Tuberculosis Onset in Dialysis Patients

If you have experienced cases of tuberculosis onset in dialysis patients, please share any difficulties encountered.

Supplemental Digital Content 2. An overview of dialysis facilities that responded to the survey

Regional distribution

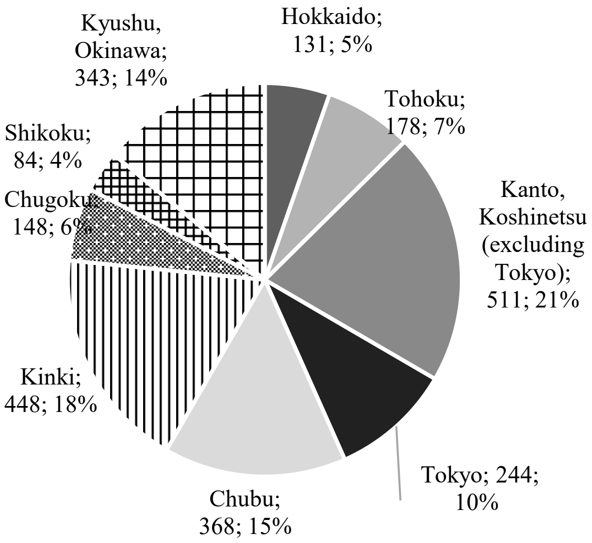

Number of responses: 2,455

Types of facilities

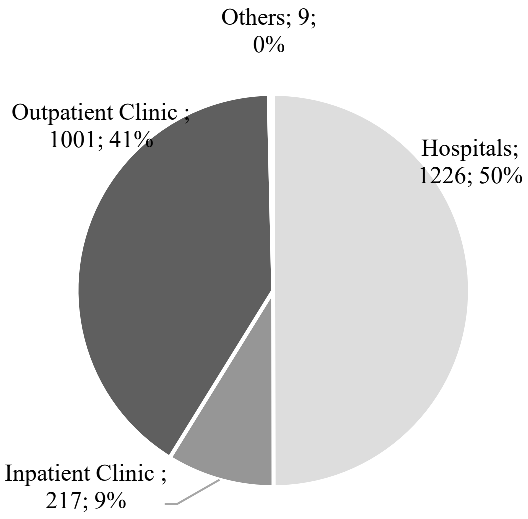

Number of responses: 2,468

Number of patients on maintenance dialysis at each facility (/patients): 66.0 (IQR 37.0–106.0)
Number of available beds per dialysis session (/beds): 29.0 (IQR 19.0–41.0))
Number of available beds for isolation dialysis (/beds): 1.0 (IQR 0.0–2.0)

As of March 31, 2024

IQR, interquartile range

Supplemental Digital Content 3. Regional distribution of patients with LTBI

	Number of patients with LTBI	Number of patients with LTBI, per facility	%
Hokkaido	9	0.069	2.8
Tohoku	17	0.096	5.2
Kanto, Koshinetsu (excluding Tokyo)	67	0.131	20.5
Tokyo	49	0.202	15.0
Chubu	40	0.109	12.2
Kinki	58	0.129	17.7
Chugoku	19	0.129	5.8
Shikoku	4	0.048	1.2
Kyushu, Okinawa	64	0.188	19.6

Number of responses: 331

LTBI, latent tuberculosis infection

Supplemental Digital Content 4. Factors influencing diagnosis of LTBI

		exp(B)*	95%C.I. lower	95%C.I. upper	p-value
Region distribution	Shikoku (ref.)	1.000			
	Tohoku	1.135	0.565	2.279	0.722
	Hokkaido	1.144	0.552	2.373	0.717
	Kanto, Koshinetsu (excluding Tokyo)	1.422	0.764	2.645	0.267
	Kyusyu, Okinawa	1.447	0.767	2.733	0.254
	Kinki	1.511	0.812	2.815	0.193
	Chubu	1.544	0.823	2.894	0.176
	Chugoku	1.620	0.809	3.243	0.173
	Tokyo	2.001	1.040	3.848	0.038
Facility type	Hospital (ref.)	1.000			
	Clinic	0.579	0.469	0.716	<0.001
Number of dialysis patients	< 50(ref.)	1.000			
	50 ≦＜90	0.709	0.550	0.914	0.008
	90 ≦	0.987	0.775	1.257	0.915

Number of analysis: 2,346

*: The exponentiated coefficients (exp(B)) represent the estimated rate ratios, with the reference category assigned a value of 1.

Supplemental Digital Content 5. Regional distribution of patients with active TB

	Number of patients with active TB	Number of patients with active TB, per facility	%
Hokkaido	7	0.053	3.6
Tohoku	13	0.073	6.6
Kanto, Koshinetsu (excluding Tokyo)	40	0.078	20.4
Tokyo	26	0.107	13.3
Chubu	20	0.054	10.2
Kinki	44	0.098	22.4
Chugoku	14	0.095	7.1
Shikoku	7	0.083	3.6
Kyushu, Okinawa	25	0.074	12.8

TB, tuberculosis

Supplemental Digital Content 6. Factors influencing the diagnosis of active TB

		exp(B)*	95%C.I.		p-value
			lower	upper	
Region distribution	Chubu (ref.)	1.000			
	Hokkaido	1.214	0.506	2.913	0.664
	Kanto, Koshinetsu (excluding Tokyo)	1.490	0.844	2.630	0.169
	Kyusyu, Okinawa	1.581	0.852	2.933	0.146
	Tohoku	1.604	0.785	3.278	0.195
	Sikoku	1.612	0.673	3.862	0.284
	Chugoku	1.657	0.798	3.442	0.176
	Kinki	2.053	1.179	3.576	0.011
	Tokyo	2.897	1.572	5.340	0.001
Facility type	Hospital (ref.)	1.000			
	Clinic	0.528	0.389	0.717	<0.001
Number of dialysis patients	< 50(ref.)	1.000			
	50 ≦<90	0.490	0.314	0.763	0.002
	90 ≦	0.549	0.377	0.799	0.002

Number of analysis: 2,308

*: The exponentiated coefficients (exp(B)) represent the estimated rate ratios, with the reference category assigned a value of 1.

Supplemental Digital Content 7. Comparison of age-specific tuberculosis incidence rates between the general population and dialysis patients

Age Group	TB Incidence in Dialysis Patients(per 100,000 PY, 95% CI)	TB Incidence in General Japanese Population (per 100,000)
<50 years	27.1 (7.1 – 47.1)	17.44
50–59	21.4 (10.3 – 32.5)	4.35
60–69	36.6 (24.2 – 49.0)	5.76
70–79	60.0 (48.5 – 71.5)	11.87
80–89	63.7 (48.0 – 79.4)	30.11
≥90	112.8 (62.3 – 163.3)	53.43
